# MEPicides: potent antimalarial prodrugs targeting isoprenoid biosynthesis

**DOI:** 10.1038/s41598-017-07159-y

**Published:** 2017-08-21

**Authors:** Rachel L. Edwards, Robert C. Brothers, Xu Wang, Maxim I. Maron, Peter D. Ziniel, Patricia S. Tsang, Thomas E. Kraft, Paul W. Hruz, Kim C. Williamson, Cynthia S. Dowd, Audrey R. Odom John

**Affiliations:** 1 Department of Pediatrics, Washington University School of Medicine, St. Louis, MO USA; 2 Department of Chemistry, George Washington University, Washington, DC USA; 3 Department of Biology, Loyola University Chicago, Chicago, IL USA; 4 Uniformed Services University of the Health Sciences, Bethesda, MD USA; 5 Tuberculosis Research Section, Laboratory of Clinical Infectious Diseases, NIAID, NIH, Bethesda, MD USA; 6 Department of Cell Biology and Physiology, Washington University School of Medicine, St. Louis, MO USA; 7 Department of Molecular Microbiology, Washington University School of Medicine, St. Louis, MO USA; 8 Present Address: Albert Einstein College of Medicine, Bronx, New York USA; 9 Roche Pharma Research and Early Development, Roche Innovation Center, Munich, Nonnenwald, Penzberg Germany

## Abstract

The emergence of *Plasmodium falciparum* resistant to frontline therapeutics has prompted efforts to identify and validate agents with novel mechanisms of action. MEPicides represent a new class of antimalarials that inhibit enzymes of the methylerythritol phosphate (MEP) pathway of isoprenoid biosynthesis, including the clinically validated target, deoxyxylulose phosphate reductoisomerase (Dxr). Here we describe RCB-185, a lipophilic prodrug with nanomolar activity against asexual parasites. Growth of *P. falciparum* treated with RCB-185 was rescued by isoprenoid precursor supplementation, and treatment substantially reduced metabolite levels downstream of the Dxr enzyme. In addition, parasites that produced higher levels of the Dxr substrate were resistant to RCB-185. Notably, environmental isolates resistant to current therapies remained sensitive to RCB-185, the compound effectively treated sexually-committed parasites, and was both safe and efficacious in malaria-infected mice. Collectively, our data demonstrate that RCB-185 potently and selectively inhibits Dxr in *P. falciparum*, and represents a promising lead compound for further drug development.

## Introduction

Despite intense efforts in drug development and aggressive vector control programs, malaria remains a formidable challenge to public health. According to recent estimates, malaria causes 212 million clinical cases and 429,000 deaths each year, predominately in young children living in sub-Saharan Africa^1^. While five species of apicomplexan parasites of the genus *Plasmodium* cause human malaria, *Plasmodium falciparum* is the most deadly^1^. Due to pervasive drug resistance, *P. falciparum* treatment has become increasingly dependent on a single class of compounds, the artemisinins. Substantial evidence suggests that the effectiveness of artemisinin-based combination therapies (ACTs) is waning, thus threatening global malaria control^2–4^. Novel, clinically validated drug targets that can be exploited for target-based optimization are urgently needed.

The methylerythritol phosphate (MEP) pathway of isoprenoid biosynthesis is a well validated but unexploited drug target present in most eubacteria and apicomplexan protozoa. In *P. falciparum*, the MEP pathway enzymes are apicoplast-localized, and data suggest that isoprenoid precursor biosynthesis may be the only essential function of the plastid organelle in blood stage parasites^5, 6^. The pathway is initiated by the condensation of pyruvate and glyceraldehyde-3-phosphate, and proceeds through a series of enzymatic reactions to produce isopentenyl pyrophosphate (IPP) and dimethylallyl diphosphate (DMAPP), which are used to synthesize downstream products. The enzymes of the MEP pathway are essential, as isoprenoids are required for numerous cellular processes including aerobic respiration, membrane stability, and protein prenylation^7, 8^. Importantly, humans employ an alternate route for isoprenoid generation, using instead the mevalonate pathway whose components lack similarity to MEP pathway enzymes. Due to the essentiality of the MEP pathway in *P. falciparum* and the absence of mammalian homologs, compounds that specifically inhibit MEP pathway enzymes are highly desirable.

The first committed enzymatic reaction of the MEP pathway is catalyzed by 1-deoxy-D-xylulose-5-phosphate reductoisomerase (Dxr/IspC; E.C. 1.1.1.267), and considerable efforts have been made to effectively target this enzyme^9–11^. Dxr catalyzes the reductive isomerization of 1-deoxy-D-xylulose 5-phosphate (DXP) to 2-C-methyl-D-erythritol 3-phosphate (MEP), using a divalent cation (Mg^2+^, Mn^2+^, or Co^2+^) and NADPH as a cofactor^12^. Chemical inhibition of Dxr in blood stage *P. falciparum* depletes cellular MEP metabolites, and ultimately kills the parasites^13^. Further, Dxr is druggable, contains a high flux-control coefficient, and is one of only seven antimalarial targets that have been clinically validated^9, 12, 14, 15^. These data demonstrate the essentiality of the Dxr enzyme and its value as a therapeutic target to combat *P. falciparum* malaria.

**Figure 1:**
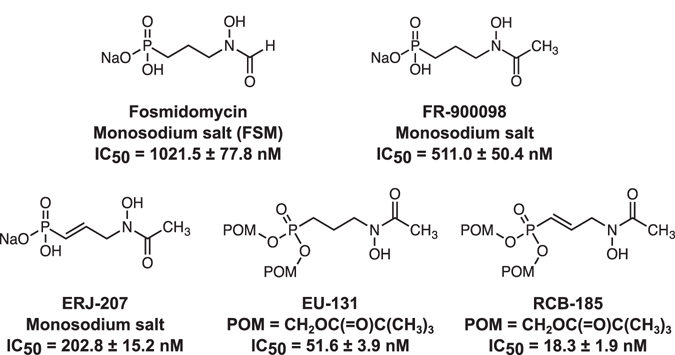
Dxr inhibitors tested against *P. falciparum*. Displayed are the structures and IC_50_ values (mean ± SEM) against *P. falciparum* strain 3D7 from 9 or more independent experiments.

The best-characterized antimalarial agent known to target Dxr is the phosphonic acid antibiotic fosmidomycin (FSM, FR-31564) (Fig. 1), a slow, tight-binding inhibitor with two modes of inhibition (competitive and noncompetitive)^16–20^. Data indicate that FSM directly inhibits the *P. falciparum* Dxr enzyme with a half-maximal inhibitory concentration (IC_50_) of 21–160 nM, and FSM is active against asexual *P. falciparum* ^21^. Since the MEP pathway is absent in mammals, FSM is safe, with mouse and rat LD_50_ values of >11,000 mg/kg following oral administration^22^. Similarly, FSM is well tolerated in human patients^23–26^.

Unfortunately, the potency of FSM against whole parasites is relatively poor (published IC_50_ values range between 0.4 to 3.7 μM), and parasite clearance is slow with a mean clearance time of 44 ± 18 h (mean ± SD; clearance defined as the time from treatment initiation until the first of two negative blood smears)^21, 24^. FSM also demonstrates several unfavorable pharmacokinetic properties, including a short serum half-life (1.87 h) and poor oral bioavailability (20–40%)^23, 27^. In addition, a substantial proportion of patients treated with FSM, in either monotherapy or in combination, suffer from recrudescent infections, likely due to suboptimal drug characteristics^24, 28^. Despite these shortcomings, FSM partnered with piperaquine has been evaluated in Phase II clinical trials as a non-artemisinin-based combination therapy to treat acute *P. falciparum* malaria^29, 30^.

To build on its inherent safety and efficacy, but improve upon its potency and pharmacokinetics, we examined a series of compounds structurally-related to FSM^31, 32^. These compounds differ structurally from FSM in the degree of unsaturation, terminal acetamide and/or presence of a lipophilic diester. The diester, often used as a cleavable prodrug, is putatively cleaved, yielding the active phosphonate inhibitor^10, 33, 34^. Since these compounds were fashioned to specifically target the Dxr enzyme of the MEP pathway, we have termed members of this series MEPicides. The most active antimalarial MEPicide was RCB-185, bearing three structural differences compared with FSM: an unsaturated propylene linker, a terminal acetamide, and a dipivaloyloxymethyl (diPOM) ester (Fig. 1). In this work, we find that RCB-185 is a potent antimalarial agent that kills parasites through MEP pathway inhibition intracellularly, and effectively treats malaria in mice. Taken together, our work demonstrates the antimalarial potential of RCB-185, and supports exploration of novel MEPicides as therapeutic agents.

## Results

### The MEPicide RCB-185 is a potent and specific inhibitor of asexual *P. falciparum* parasites

To evaluate the activity of the Dxr inhibitors against blood stage parasites, we treated asynchronous cultures of *P. falciparum* strain 3D7 with EU-131, ERJ-207, or RCB-185, and then quantified growth after 72 h^35^. While both EU-131 and ERJ-207 were active against asexual parasites (51.6 ± 3.9 nM and 202.8 ± 15.2 nM, respectively), RCB-185 was the most potent compound with a mean half-maximal inhibitory concentration (IC_50_) = 18.3 ± 1.9 nM (Fig. 1 and Table 1). For comparison, the *in vitro* activity of RCB-185 is similar to that of the current first-line antimalarial agent artemisinin, which has an IC_50_ = 10.4 ± 1.6 nM against 3D7 parasites (mean ± SEM from >3 independent experiments, data not shown). Furthermore, RCB-185 has a 50-fold improved IC_50_ value compared with the well-described Dxr inhibitor FSM [IC_50_ value of 1021.5 ± 77.8 nM (Fig. 1 and Table 1)] and is 28-fold more potent than the acetyl derivative of FSM, FR-900098 [IC_50_ value of 511.0 ± 50.4 nM (Fig. 1 and Supplementary Table S1)]. These data indicate that RCB-185 is a robust inhibitor of asexual parasite growth.

**Table 1:** RCB-185 is active against multidrug resistant *P. falciparum*.

Cell line	FSM IC_50_ (nM)	RCB-185 IC_50_ (nM)
*P. falciparum* 3D7 (pan-sensitive, lab adapted)	1021.5 ± 77.8	18.3 ± 1.9
*P. falciparum* 7G8 (quinine, chloroquine, and pyrimethamine resistant)	1235.7 ± 188.7	46.7 ± 10.9
*P. falciparum* D6 (mefloquine resistant)	1339.5 ± 246.7	37.6 ± 10.0
*P. falciparum* D10 (mefloquine resistant)	2184.0 ± 353.0	42.5 ± 11.5
*P. falciparum* K1 (chloroquine and sulfadoxine-pyrimethamine resistant)	837.0 ± 141.0	31.9 ± 1.0
*P. falciparum* IPC 5202 (chloroquine and artemisinin resistant)	862.8 ± 247.5	21.1 ± 1.8
Human HepG2 cells	n.d.	>100,000

Since our data indicate that RCB-185 is substantially more active than FSM, we performed molecular docking experiments to understand how RCB-185 might have distinct interactions with the target enzyme Dxr. We docked ERJ-207, the active component of RCB-185, to the *P. falciparum* Dxr crystal structure (PDB 3AU8) in the apo form with NADPH and Mn^2+^ bound^36^. All predicted binding conformations of ERJ-207 interact with Dxr at the DOXP binding cavity, and three dominant conformations for ERJ-207 were observed (Fig. S1). These orientations were similar to the conformations observed for FSM, and thus a unique binding mode appears unlikely to account for the potency of RCB-185^37^.

### RCB-185 inhibits isoprenoid biosynthesis in *P. falciparum*

**Figure 2:**
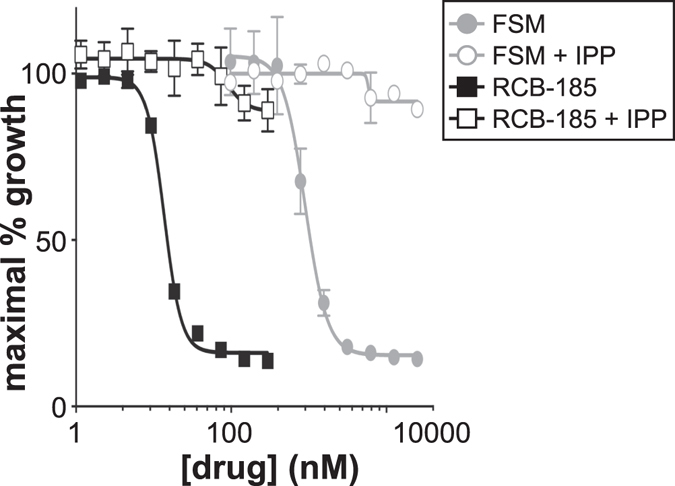
Isoprenoid precursors rescue RCB-185-treated *P. falciparum*. *P. falciparum* strain 3D7 was treated with FSM (grey circles) or RCB-185 (black squares) at a range of concentrations and growth quantified by PicoGreen (Life Technologies) after 72 h, as previously described^35^. The downstream isoprenoid precursor IPP rescues both FSM and RCB-185-treated parasites (open shapes) indicating the fosmidomycin analog is a specific inhibitor of the MEP pathway in *P. falciparum*. Shown is a representative graph from 3 or more independent experiments.

Malaria parasites treated with small molecules that specifically target apicoplast functions, including compounds that block MEP pathway enzymes, are chemically rescued if cultures are supplemented with the isoprenoid precursor IPP^5, 38^. To investigate whether the growth inhibition caused by MEPicide treatment is due to inhibition of IPP synthesis, we treated *P. falciparum* with compounds over a range of concentrations (1.2 nM–4606.4 nM) and evaluated for IPP-mediated growth restoration. As displayed in Fig. 2, 250 μM IPP supports growth of *P. falciparum* treated with RCB-185 at all concentrations tested. These data are similar to the chemical complementation observed in FSM- and FR-900098-treated parasites supplemented with IPP (Fig. 2; previously reported in ref. 5; Fig. S2). In addition, growth of *P. falciparum* treated with the less potent FSM-like compounds, EU-131 and ERJ-207, was also rescued by IPP supplementation (Fig. S2).

**Figure 3:**
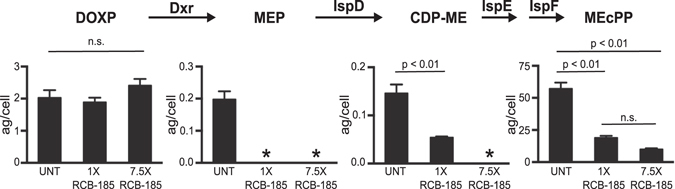
RCB-185 inhibits the MEP pathway in *P. falciparum*. MEP pathway metabolites were compared between untreated (UNT) *P. falciparum* and parasites treated with RCB-185 at either 1x or 7.5x the WT IC_50_ value of 18.3 nM. After 10 h treatment, cultures were saponin-lysed, and the pellets were analyzed by LC-MS/MS. Displayed are the means ± SEM of the metabolite levels from 3 independent experiments. No statistically significant difference was denoted by n.s., and asterisks indicate metabolites that were below the limit of detection (CDP-ME below 1.25 ng/mL and MEP below 12.5 ng/mL).

The chemical rescue of RCB-185-treated parasites indicates that the compound affects an apicoplast function, and strongly implicates isoprenoid biosynthesis as the specific target. As RCB-185 is an analog of FSM, we predicted that treatment of *P. falciparum* with the MEPicide would deplete pathway intermediates beyond its proposed target, Dxr. To directly quantify MEP metabolites from treated parasites, we employed a liquid chromatography-mass spectrometry (LC-MS) method that measures cellular levels of key MEP pathway intermediates, including 1-deoxy-D-xylulose 5-phosphate (DOXP), 2-C-methylerythritol 4-phosphate (MEP), 4-diphosphocytidyl-2-C-methylerythritol (CDP-ME) and 2-C-methyl-D-erythritol 2,4-cyclopyrophosphate (MEcPP)^13^. Briefly, 3D7 parasites were treated + /- RCB-185 for 10 h and MEP pathway metabolite levels were measured by LC-MS/MS. As anticipated, our metabolic profiling data indicated there was no significant difference in the levels of DOXP, the substrate for *P. falciparum* Dxr, in RCB-185-treated cultures (Fig. 3). However, MEP pathway intermediates measured downstream of Dxr were greatly diminished in treated parasites. Both MEP and CDP-ME were below the limits of detection (asterisks; 12.5 ng/mL and 1.25 ng/mL, respectively)^13^, and levels of the most distal metabolite, MEcPP, were significantly reduced when compared to untreated parasites (p < 0.01) (Fig. 3). Specifically, *P. falciparum* treated with RCB-185 at 1x and 7.5x the IC_50_ value of 18.3 nM corresponded to 3.0- and 5.7-fold reductions in MEcPP levels, respectively. Our metabolic data strongly suggest that RCB-185 inhibits the first committed step of isoprenoid biosynthesis through direct inhibition of Dxr.

**Figure 4:**
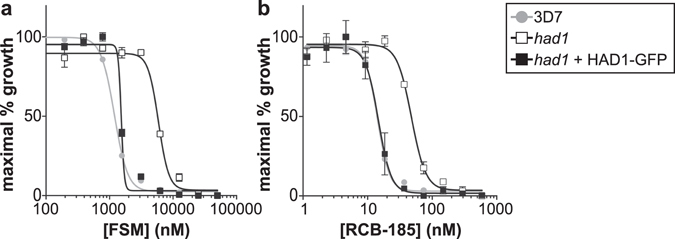
*P. falciparum* strains with high levels of the Dxr substrate DOXP confer RCB-185 resistance. Dose-dependent growth inhibition by FSM (**a**) or RCB-185 (**b**) was determined for the parental strain (3D7; closed circles, grey line), the FSM-resistant PfHad1 loss-of-function parasite strain (*had1*; open squares, black line), and the FSM-sensitive complemented strain (*had1* + HAD1-GFP; closed squares, black line) as previously described^35^. For FSM, the parent strain has an IC_50_ = 1021.5 ± 77.8 nM, while *had1* has an IC_50_ = 5348.1 ± 830.6 nM. For RCB-185, the parent strain has an IC_50_ = 18.3 ± 1.9 nM, while *had1* has an IC_50_ = 76.5 ± 18.5 nM. Data (mean ± SEM) are representative of at least 3 independent biological replicates performed in duplicate.

To further interrogate whether our FSM-like compounds inhibit the MEP pathway, we evaluated its antimalarial efficacy against a *P. falciparum* strain that produces high levels of the Dxr substrate, DOXP, due to a mutation in the metabolic regulator *HAD1* (PF3D7_1033400). Previous data indicate that an increase in the intracellular pool of DOXP impedes the competitive inhibition of Dxr by FSM, which results in FSM-resistant parasites^39^. We predicted that a surplus in cellular DOXP would promote MEPicide resistance by a mechanism analogous to the FSM and FR-900098 resistance displayed by *had1* parasites (*had1* parasites were 5.2- and 4.2-fold more resistant to FSM and FR-900098, respectively; Figs 4a and S3)^39^. Indeed, *had1* parasites were 4.2-fold more resistant to RCB-185 with an IC_50_ = 76.5 ± 18.5 nM when compared to an IC_50_ = 18.3 ± 1.9 nM for WT parasites (p < 0.05, Fig. 4b). Importantly, RCB-185 sensitivity was restored if a WT copy of *HAD1* was supplied in the mutant strain (IC_50_ = 26.0 ± 6.4; Fig. 4b). Similar results were obtained with EU-131 and ERJ-207, such that *had1* parasites were more resistant to the compounds than WT parasites (Fig. S3; *had1* parasites were 8.0- and 3.3-fold more resistant to EU-131 and ERJ-207, respectively). These data indicate that, similar to FSM, MEPicides likely compete with cellular DOXP. Collectively, our results demonstrate that our novel inhibitors specifically inhibit *P. falciparum* growth through competitive inhibition of Dxr of the isoprenoid biosynthetic pathway. In particular, our data validate that the most potent compound RCB-185 elicits antimalarial activity via Dxr inhibition, without significant off-target toxicities.

### RCB-185 is active against drug-resistant *P. falciparum*

A major threat to public health is the emergence of *P. falciparum* resistance to current therapies, including the frontline agents artemisinin and its derivatives artesunate and artemether^40^. Thus, the discovery and development of new classes of antibiotics that maintain effectiveness against drug-resistant parasites is essential. To assess whether RCB-185 inhibits growth of resistant parasites, we obtained clinical isolates of *P. falciparum* that were resistant to quinine, chloroquine, mefloquine, sulfadoxine/pyrimethamine, and/or artemisinin. The isolates were treated with RCB-185 for 72 h and growth inhibition quantified as described^35^. As displayed in Table 1, the resistant strains were sensitive to RCB-185, and the IC_50_ values were only modestly different than WT (3D7) parasites [IC_50_ shift between WT and resistant strains ≤3-fold;^41^]. These data demonstrate the potential utility of MEPicides in combating drug-resistant parasite strains.

### RCB-185 is active against *P. falciparum* parasites committed to gametocytogenesis

**Figure 5:**
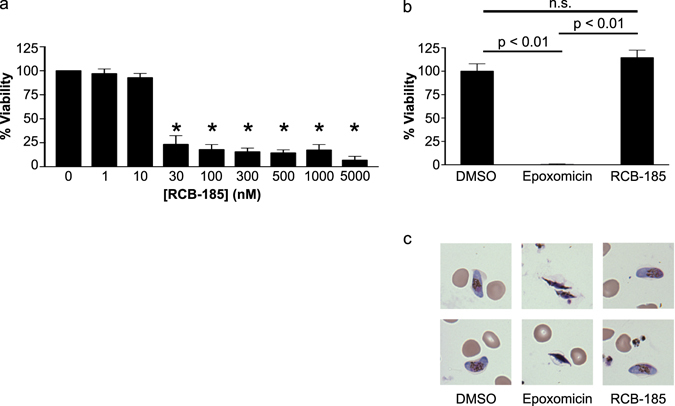
RCB-185 is active against sexually-committed *P. falciparum* parasites. (**a**) Gametocyte-committed trophozoites and schizonts were treated with RCB-185 (1 nM–5 uM) for 72 h and viability assessed by flow cytometry using the mitochondrial dye MitoProbe DiIC(1)5. Displayed are the means ± SEM from at least 2 independent biological replicates. Asterisks denote RCB-185 treatments that were statistically different than untreated parasites (p < 0.01). (**b**) Stage III/IV/V gametocytes were purified on a Percoll gradient and then treated with 5 μM RCB-185 for 72 h. Parasites were stained with MitoProbe DiIC(1)5 and viability quantified by flow cytometry. 32.5 nM epoxomicin and 0.5% DMSO were used as positive and negative controls, respectively. While the well-characterized antimalarial agent epoxomicin efficiently kills mature gametocytes (p < 0.01), flow cytometry data indicate that RCB-185 lacks activity against mature gametocytes. Displayed are the means ± SEM from at least 2 independent biological replicates (**c**) Representative Giemsa-stained blood smears of parasites treated in (**b**) demonstrate that 5 μM RCB-185 is inactive against stage III/IV/IV gametocytes.

Antimalarial development efforts prioritize compounds that target a broad range of life cycle stages, including the sexual gametocyte stages of the parasite required for mosquito transmission^42^. Previous studies of the MEP pathway in *P. falciparum* gametocytes indicate that MEP pathway intermediates are present and isoprenoid biosynthesis appears to be required for gametocyte development^43^. However, FSM has been found to be ineffective against gametocytes in multiple independent studies^24, 44–46^. To assess whether RCB-185 could inhibit growth of parasites committed to gametocytogenesis, asynchronous cultures were treated with *N*-acetylglucosamine (NAG), which eliminates asexually replicating *P. falciparum*. The sexually-committed parasites were then treated with RCB-185 for 72 h at a range of concentrations (1 nM–5 μM). Parasites were stained with a mitochondrial membrane potential dye [MitoProbe DiIC(1)5] and viability assessed by flow cytometry. As displayed in Fig. 5a, trophozoite and schizont stage parasites committed to gametocytogenesis were susceptible to RCB-185, as treatments as low as 30 nM caused a drastic reduction in viability (4.3-fold reduction). To evaluate whether the MEPicides could inhibit mid-stage (II/III) or late-stage (III-V) gametocytes, we purified *P. falciparum* gametocytes and then treated the cells with compounds for 72 h (1 nM–5 μM). Parasites were stained and viability measured as before. Unlike the known gametocytocidal agent epoxomicin, MEPicides were inactive against stage II-V gametocytes, even at concentrations >250x the asexual IC_50_ values (Fig. 5b, Figs S4 and S5). Consistent with the flow cytometry data, Giemsa-stained blood smears confirmed that epoxomicin dramatically alters the integrity of late-stage gametocytes, whereas RCB-185-treated gametocytes were unchanged (Fig. 5c and data not shown).

### RCB-185 is potent, safe, and efficacious in a mouse model of malaria

**Figure 6:**
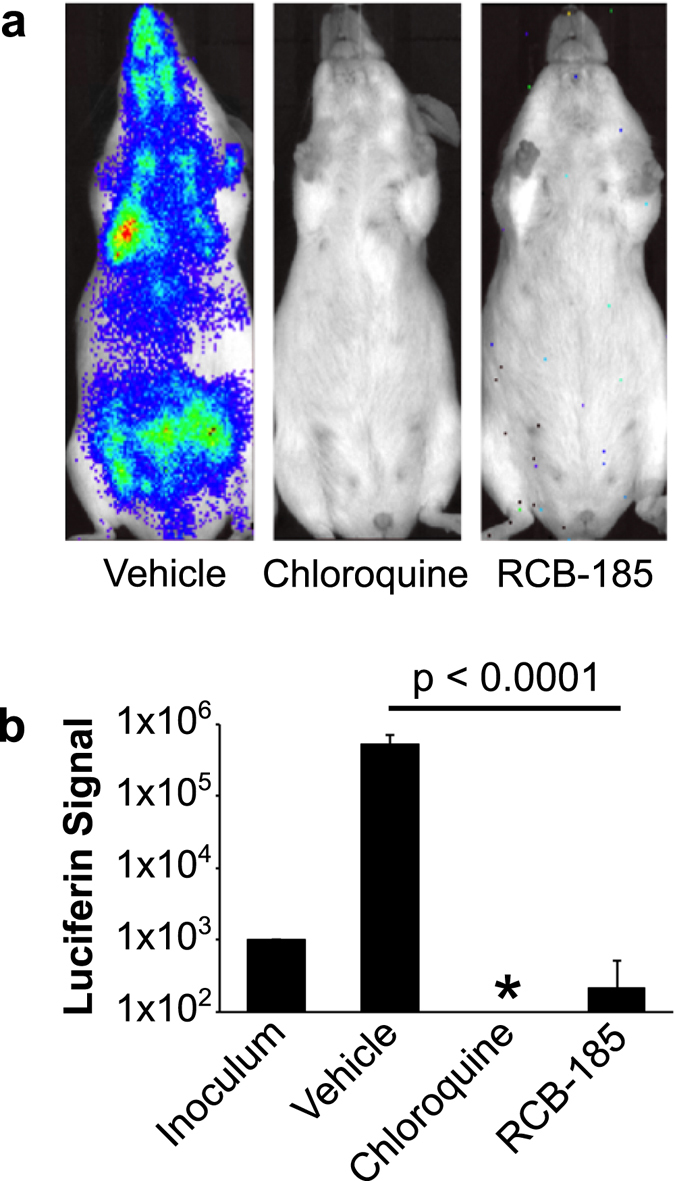
RCB-185 is efficacious in a mouse model of malaria. Groups of 5-7 Swiss Webster mice were infected with 10^3^ *P. berghei* ANKA expressing luciferase via i.p. injection. On days 2–7, mice were dosed once per day with vehicle, 20 mg/kg chloroquine, or 50 mg/kg RCB-185. At 7 days post-infection, mice were imaged with an IVIS imager and the parasite burden quantified by luciferase signal intensity. Representative images are shown for each treatment condition (top), and the average luciferase signal intensity quantified for each treatment (bottom). Displayed are the means ± SD for each treatment group. The asterisk indicates the luciferase signal was below the limit of detection.

Due to the potent antimalarial activity of RCB-185 against asexual *P. falciparum in vitro*, we then tested whether the compound could effectively treat asexual blood stages in a mouse model of malaria. Briefly, groups of female Swiss Webster mice were infected by intraperitoneal (i.p.) injection with 10^3^ blood-stage *P. berghei* ANKA expressing luciferase. After two days post-infection, mice were dosed daily with vehicle, 20 mg/kg chloroquine, or 50 mg/kg RCB-185. Mice were treated for 5 days, and the parasite burden was quantified by measuring the luciferase signal intensity at 7 days post-infection. As demonstrated by the vehicle control, the number of *P. berghei* parasites expands by several orders of magnitude after one week of infection (Fig. 6). Parasite numbers were dramatically reduced in mice administered the control therapeutic chloroquine, as the luciferase signal was below the limit of detection (designated by an asterisk). Notably, *P. berghei*-infected mice treated with RCB-185 had more than a 3-log drop in the parasite load when compared to the vehicle (p < 0.0001; Fig. 6). In addition, no adverse effects were observed in mice administered the MEPicide, suggesting the compound was well tolerated at the treatment concentration. This corroborates preliminary data that indicates RCB-185 is nontoxic to human cell lines (Table 1; HepG2 cells >100,000 nM) with a selectivity index >5000 (selectivity index calculated as the HepG2 cell IC_50_/*P. falciparum* 3D7 IC_50_), and thus, is a valuable antimalarial agent^47^.

## Discussion

New antimalarials are urgently needed, and PfDxr represents a promising essential, druggable, and parasite-specific target for antimalarial drug development. The well-characterized Dxr inhibitor FSM exhibits poor antimalarial potency and sub-par pharmacokinetic and pharmacodynamic properties^23–25, 27, 28^. Nevertheless, the successful use of FSM to treat uncomplicated malaria infections suggests that improved Dxr-targeting agents are likely to be of extremely high value^24, 25^. Here, we report the discovery and characterization of RCB-185, a potent and specific MEPicide antimalarial that targets *P. falciparum* Dxr. In our hands, the activity of RCB-185 (IC_50_ = 18.3 ± 1.9 nM; Table 1) rivals that of current first-line therapeutics (artemisinin IC_50_ = 10.4 ± 1.6 nM; chloroquine IC_50_ = 14.5 ± 0.9 nM; data not shown).

The MEPicide family of compounds were designed to inhibit bacterial and parasitic growth by targeting Dxr. Evidence strongly supports that RCB-185, and the related MEPicides EU-131 and ERJ-207, inhibit intracellular *P. falciparum* Dxr as predicted. First, growth of MEPicide-treated parasites is rescued by IPP supplementation, suggesting that the compounds inhibit the isoprenoid biosynthetic pathway and lack additional modes of action (Figs 2 and S2). Second, targeted metabolite analysis is consistent with Dxr inhibition, as metabolites beyond the proposed block are drastically reduced (Fig. 3). Finally, our data indicate *P. falciparum* parasites with elevated levels of the isoprenoid precursors, including DOXP, are more resistant to MEPicide treatment, as previously reported for FSM (Figs 4 and S3)^39^. Our data strongly suggest that, like FSM, MEPicides are competitive with the Dxr substrate, DOXP. Altogether, our studies demonstrate that these FSM-like compounds inhibit *P. falciparum* through direct Dxr inhibition, without substantial off-target effects, and that further investigation of the most active compound, RCB-185, as a novel antimalarial is justified. Importantly, having a therapy with a known, parasite-specific, and proven mechanism-of-action will facilitate future efforts to develop similarly structured analogs.

*P. falciparum* has developed resistance to all clinically used agents including the aryl aminoalcohols (quinine), aminoquinolines (chloroquine), aminoalcohols (mefloquine), antifolates (sulfadoxine and pyrimethamine), and endoperoxides (artemisinin)^48^. To subvert the development of resistance and augment the therapeutic lifetime of an antimalarial drug, entities should be chemically distinct and target novel cellular processes^48^. RCB-185 is based on a unique chemical scaffold, and the Dxr enzyme is not targeted by existing therapies. We expected that *P. falciparum* strains that are resistant to a wide range of antimalarial compounds would be susceptible to RCB-185. To this end, we tested RCB-185 against a battery of drug-resistant parasites. As predicted, no meaningful shifts in the IC_50_ values were detected between 3D7 and resistant *P. falciparum* treated with either FSM of RCB-185 (Table 1; clinically relevant IC_50_ shift ≥ 3-fold,^41^). These data demonstrate that RCB-185 may be an effective tool for combating environmental isolates refractory to current treatments.

Data indicate that gametocytes contain detectable levels of MEP pathway intermediates, and isoprenoid products are essential for gametocytogenesis^43, 49^. Accordingly, the MEP pathway enzymes are promising targets for eliminating the sexual-stages of *P. falciparum*. Previous studies have shown that FSM lacks gametocytocidal activity^44, 46, 50^. We similarly found that the MEPicides were inactive against stage II-V gametocytes and suspect this may be due to defects in their cellular import^51^ (Fig. 5, Figs S4 and S5). However, our data suggest that RCB-185 is active against sexually-committed trophozoites and schizonts, indicating that this novel antimalarial agent will have modest transmission-blocking activity (Fig. 5A). We anticipate that improving anti-gametocytocidal activity will remain an important goal during further development of the MEPicides^44^.

While a number of FSM-inspired compounds have potent activity against *P. falciparum* grown in culture, few Dxr inhibitors have moved beyond preliminary *in vivo* evaluation^52–58^. Brücher *et al*. previously identified FSM derivatives that were potent against asexual parasites but disappointingly lacked *in vivo* efficacy in both the *P. berghei* and *P. falciparum* SCID mouse models of disease^21^. In contrast, RCB-185 represents a significant advance over prior Dxr inhibitors, as a superior agent both highly potent against cultured *P. falciparum*, as well as therapeutically effective in treatment of *P. berghei*-infected mice, with a dramatic reduction of viable parasites (>3 log reduction; Fig. 6). We speculate that the lipophilic moiety present in our compounds may aid in erythrocyte and/or parasite membrane penetration or may prolong the plasma retention time of the compounds, which ultimately enhances their antimalarial activity.

Prior studies indicate that FSM and its acetyl derivative FR-900098 are well tolerated in rats and mice at high doses^20, 22^. In addition, several reports have shown that FSM is safe in human patients, and FSM is currently under evaluation in Phase II clinical trials^23–26, 29^. Since many structural features of RCB-185 are similar to FSM, we predict that analogs of RCB-185 will be safe for treating acute *P. falciparum* malaria. Our preliminary studies indicate minimal cellular toxicity and no grossly apparent toxicity in mice. Additional studies will be needed to carefully evaluate the safety profile of RCB-185, and likewise, assess the pharmacokinetic and pharmacodynamic properties of this novel antimalarial.

Many important human pathogens employ the MEP pathway to synthesize isoprenoids for essential cellular functions, including *Toxoplasma gondii*, *Mycobacterium tuberculosis*, *Escherichia coli*, *Yersinia pestis*, and *Pseudomonas aeruginosa*. Due to the conservation of the MEP pathway enzymes, in particular Dxr, we anticipate that the MEPicides will have broad-spectrum activity. Indeed, we have previously found that RCB-185 has activity against *M. tuberculosis* ^31^. Additional studies will be required to discern whether drug-like properties of the MEPicides, including penetration and efflux, can be enhanced to improve potency against a large array of pathogenic organisms.

## Materials and Methods

### *P. falciparum* culture

*P. falciparum* strains were obtained through the MR4 as part of the BEI Resources Repository, NIAID, NIH (www.mr4.org): 3D7 (wild-type, WT), K1 (MRA-159; chloroquine and sulfadoxine-pyrimethamine resistant) deposited by D.E. Kyle^59^, 7G8 (MRA-152; quinine, chloroquine and pyrimethamine resistant) deposited by D. Walliker^60^, D6 (MRA-285; mefloquine resistant) deposited by D.E. Kyle, D10 (MRA-201; mefloquine resistant) deposited by Y. Wu, and IPC 5202 (Cam3.IR539T; MRA-1240; chloroquine and artemisinin resistant) deposited by D. Ménard. A *P. falciparum* strain containing increased levels of MEP pathway metabolites, *had1* (MRA-1257), and its isogenic complement, *had1* + PfHad1-GFP (MRA-1258), were generated in strain 3D7, as reported^39^. Parasites were cultured in a 2% suspension of human erythrocytes and RPMI 1640 (Sigma) medium supplemented with 27 mM sodium bicarbonate, 11 mM glucose, 5 mM HEPES, 1 mM sodium pyruvate, 0.37 mM hypoxanthine, 0.01 mM thymidine, 10 µg/mL gentamicin, and 0.5% Albumax (Gibco) at 37 °C, 5% O_2_/5% CO_2_/90% N_2_ atmosphere as previously described^13, 61^.

### EU-131, ERJ-207, and RCB-185

EU-131 and ERJ-207 were prepared as described^31, 32^. RCB-185 was prepared using the method described previously^31^ with one modification. The final step is a debenzylation reaction using boron trichloride to afford the target molecule, but at a particularly low yield (13%). We improved this reaction with the addition of radical scavenger pentamethylbenzene^62^ and the yield improved significantly to 50% (Fig. S6).

### *P. falciparum* growth inhibition assays

Asynchronous *P. falciparum* cultures were diluted to 1% parasitemia and treated with inhibitors at concentrations ranging from 0.25 ng/mL–100 μg/mL. Growth inhibition assays were performed in opaque 96-well plates at 100 µL culture volume. After 3 days, parasite growth was quantified by measuring DNA content using PicoGreen (Life Technologies) as described^35^. Fluorescence was measured on a FLUOstar Omega microplate reader (BMG Labtech) at 485 nm excitation and 528 nm emission. Half maximal inhibitory concentration (IC_50_) values were calculated by nonlinear regression analysis using GraphPad Prism software. For isopentenyl pyrophosphate (IPP) (Echelon) rescue experiments, 250 µM IPP was added to the appropriate wells for the duration of the experiment.

### Sample preparation for mass spectrometry analysis

*P. falciparum* strain 3D7 was cultured at 37 °C in 30 mL volumes in 100 mm tissue culture dishes (Techno Plastic Products) at 4% hematocrit until >8% parasitemia. Cultures were synchronized until >75% of parasites were in ring stage growth and then treated with RCB-185 at 1x or 7.5x the 3D7 IC_50_ for 10 h. Cultures were then lysed with 5% saponin, the parasite pellets washed with 1x phosphate-buffered saline (PBS), and the pellets stored at −80 °C. Samples were extracted via addition of 600 μL of ice-cold extraction solvent [chloroform, methanol, and acetonitrile (2:1:1, v/v/v)] and two liquid nitrogen-cooled 3.2 mm stainless steel beads, followed by homogenization in the Tissue-Lyser II instrument (Qiagen, Valencia, CA) at 20 Hz for 5 min in a cold sample rack. Ice-cold water (600 μL) was added, and samples were homogenized for an additional 5 min at 20 Hz. After centrifugation (14,000 rcf at 4 °C for 5 min), the polar upper phase was aspirated and lyophilized. Dried samples were dissolved in 100 μL water and analyzed by LC-MS/MS.

### LC-MS/MS analysis

The 4000QTRAP LC-MS/MS system (AB Sciex) was used in multiple-reaction monitoring (MRM) mode using negative ionization. The detailed instrument configuration and compound-dependent parameters for isoprenoid precursors were as previously described^13^. LC separation prior to MRM detection was achieved by ion pair reverse-phase chromatography as described previously^63^, with 10 mM tributylammonium acetate (pH 5.1-5.5) used as the ion pair reagent and the following modifications: (1) RP-hydro 100 mm × 2.0 mm, 2.5 μm high performance liquid chromatography column (Phenomenex), (2) flow rate of 0.14 mL/min, (3) solvent A of 10 mM tributylammonium acetate in 5% methanol, (4) binary LC gradient (20% solvent B (100% methanol) from 0 to 2.5 min, 30% B for 12.5 min, 80% B for 5 min, and column equilibration for 5 min), and (5) autosampler injection volume of 20 μL. For deoxyxylulose 5-phosphate (DOXP) and methylerythritol cyclodiphosphate (MEcPP) metabolites, one-way ANOVA was used to test for significance (VassarStats). A t-test was used to test for significance between UNT and 1x RCB-185 cytidine diphosphate methylerythritol (CDP-ME) levels (VassarStats). A significance test was not performed for MEP, as its levels were below the limit of detection (12.5 ng/mL) for RCB-185-treated parasites.

### Gametocyte assay

#### Cell culture

*P. falciparum* strain NF54 was grown in RPMI 1640 supplemented with 25 mM HEPES, 29 mM sodium bicarbonate (pH 7.3), 0.37 mM hypoxanthine, 5 μg/mL gentamicin (KD Biomedical, Columbia, MD), and 10% human serum (Interstate Blood Bank, Memphis, TN). To evaluate the effect of the compound on early gametocyte-committed parasites, cultures were set up at 0.2% parasitemia and 5% hematocrit and fed daily with RPMI. When the parasitemia was between 7–10%, the asynchronous culture was used directly for the drug assay described below. To isolate stage II/III gametocytes, a 3% parasitemia/6% hematocrit culture was expanded during the daily feed by dilution with additional RBCs and media to 1% parasitemia and 3% hematocrit. When the culture reached ~10% parasitemia, *N*-acetylglucosamine (NAG, 60 mM) was added for 3 days and then mid-stage gametocytes (stage II/III) were harvested by centrifugation on a 17% or 18% Nycodenz cushion (Accurate Chemical and Scientific Corporation, Westbury, NY). To isolate late-stage gametocytes (stage III-V), the gametocyte cultures were initiated at 0.2% parasitemia and 6% hematocrit. On the third day, the hematocrit was reduced to 3% by increasing the media added during the daily feed. Following NAG (50 mM) treatment on days 10–12 to eliminate asexual parasites, stage III/IV/V gametocytes were purified on a 65% Percoll gradient and returned to culture. The parasites were resuspended at 10% gametocytemia and 2% hematocrit the following day.

#### Drug preparation

A 96-well flat-bottom plate was prepared with 2x concentration of the test compound ranging between 10 μM to 0.002 μM in complete RPMI with 1% DMSO. Positive controls included epoxomicin (65 nM) and/or maduramicin (100 nM), while DMSO (1%) was used as a negative control. NAG was used to selectively eliminate asexually replicating parasites and to define the population of gametocyte-committed parasites. The different parasite preparations were added to individual wells at 2% hematocrit for a 1:1 dilution. The plate was gassed with 90% N_2_, 5% O_2_, 5% CO_2_, and incubated at 37 °C with compound for 72 h. Viability was assessed microscopically via Giemsa-stained thin smears and by flow cytometry as described below.

#### Flow cytometric analysis

Viability was assayed by flow cytometry using a membrane potential dye, MitoProbe DiIC1(5) [DiIC1(5); Thermo Scientific], which was previously described and validated by comparison with the oxidoreduction indicator alamarBlue^64^. Cells were resuspended at 0.1% hematocrit in 50 nM DiIC1(5) with buffer containing 1.67 mg/mL glucose, 8 mg/mL NaCl, 8 mM Tris-Cl (pH 7.4) in a 96-well V-bottom plate and incubated at 37 °C in the dark for 20 to 30 min prior to analysis on an Accuri C6 flow cytometer (BD Biosciences). Uninfected RBCs incubated with DiIC1(5) and unstained *P. falciparum* parasites were used as controls to determine the threshold for DiIC1(5) (640-nm laser excitation and FL4 emission filter [675/25 nm])-positive, single, and intact cell populations by following the gating strategy of Malleret *et al*.^65^. Viability was calculated by determining the ratio of DiIC1 (5)-positive events in drug-treated samples compared to vehicle controls. One-way ANOVA was used to test for significance between the three treatment groups (VassarStats).

#### Drug testing against *Plasmodium berghei* asexual blood stages

*In vivo* testing of RCB-185 against intraerythrocytic *P. berghei* parasites was performed by the Anti-Infectives Screening Core at NYU School of Medicine (OLAW: A3435-01). The study was carried out in strict accordance with the recommendations in the Guide for the Care and Use of Laboratory Animals of the National Institutes of Health. The protocol (140112-03) was approved by the Institutional Animal Care and Use Committee of New York University School of Medicine, which is accredited by the Association For Assessment and Accreditation of Laboratory Animal Care International (AAALAC).

Briefly, groups of 5–7 female Swiss Webster mice weighing 25–30 g were infected via intraperitoneal (i.p.) injection with 10^3^ transgenic *P. berghei* ANKA that express a fusion GFP (mutant 3) and firefly luciferase (LucIAV) fusion under the control of the constitutive eef1aα promoter that is stably integrated into the 230p locus (PbGFP-Luccon)^66^. Two days post-infection, mice were treated by i.p. injection with 50 mg/kg of the test compound RCB-185. 20 mg/kg chloroquine and 2% methyl cellulose, 0.5% tween-80 were used as positive and vehicle controls, respectively. Treatment continued once daily for 5 days. At 7 days post-infection, mice were administered 2.5% isoflurane via a nose cone, and once anesthetized, were injected i.p. with 150 mg/kg of D-Luciferin Potassium-salt (Goldbio) dissolved in PBS. Mice were imaged 5–10 min after the injection of luciferin with an IVIS 100 (Xenogen, Alameda, CA), and data acquisition and analysis were performed with LivingImage (Xenogen) to quantify the level of infection. To test for significance between vehicle- and RCB-185-treated mice, a t-test was used (VassarStats).

#### HepG2 cytotoxicity assay

HepG2 cells were cultured at 37 °C with 5% CO_2_ in Dulbecco’s modified Eagle’s medium (DMEM) containing either glucose or galactose as carbon sources. Cells were seeded onto 96-well plates at 2 × 10^4^ and allowed to adhere overnight. Compounds or controls were added in triplicate to the wells by a 2-fold serial dilution method. After 24 h, cellular ATP concentrations, which are proportional to the number of viable cells, were determined using the CellTiter-Glo Luminescent Cell Viability Assay (Promega) and the luminescence quantified via a Fluor Optima microplate reader.

## Electronic supplementary material

Supplementary Material
