# Supplementary material for: MEPicides: potent antimalarial prodrugs targeting isoprenoid biosynthesis
Source: Sci Rep. 2017 Aug 21;7:8400. doi: 10.1038/s41598-017-07159-y (PMC5567135; doi:10.1038/s41598-017-07159-y)
Supplement: Supplementary file 1 — Supplementary Material [file 41598_2017_7159_MOESM1_ESM.doc]

**Supplementary Information**

**MEPicides: potent antimalarial prodrugs targeting isoprenoid biosynthesis**

Rachel L. Edwards^1^, Robert C. Brothers^2^, Xu Wang^2^, Maxim I. Maron^3†^, Peter D. Ziniel^4^, Patricia S. Tsang^5^, Thomas E. Kraft^1†^, Paul W. Hruz^1,6^, Kim C. Williamson^3,4^, Cynthia S. Dowd^2^, Audrey R. Odom John^1,7*^

^1^Department of Pediatrics, Washington University School of Medicine, St. Louis, MO, USA, ^2^Department of Chemistry, George Washington University, Washington, DC, USA, ^3^Department of Biology, Loyola University Chicago, Chicago, IL, USA, ^4^Uniformed Services University of the Health Sciences, Bethesda, MD, USA, ^5^Tuberculosis Research Section, Laboratory of Clinical Infectious Diseases, NIAID, NIH, Bethesda, MD USA, ^6^Department of Cell Biology and Physiology, Washington University School of Medicine, St. Louis, MO, USA, ^7^Department of Molecular Microbiology, Washington University School of Medicine, St. Louis, MO, USA

^†^Present address: Maxim I. Maron, Albert Einstein College of Medicine, Bronx, New York, USA; Thomas E. Kraft, Roche Pharma Research and Early Development, Roche Innovation Center, Munich, Nonnenwald, Penzberg, Germany

^*^Address correspondence to Audrey R. Odom, [aodom@wustl.edu](mailto:odom_a@kids.wustl.edu)

**Supplementary Materials and Methods**

**Docking of compound ERJ-207 to PfDxr.** ERJ-207 was docked to PfDxr in the apo form (PDB 3AU8) ^1^ in the presence of NADPH and Mn^2+^ bound. The receptor was prepared using MGL Tools 1.5.6, and the ligand was prepared using ArgusLab 4.0.1 and MGL Tools 1.5.6. PfDxr and ERJ-207 were docked in rigid receptor, flexible ligand mode using Autodock VINA with the search space covering the entire Dxr protein ^2^. The docking was repeated three times using increasing exhaustiveness of 50, 500 and 2000 yielding comparable distributions of docked poses for each attempt. Figure S6 were generated using the PyMOL Molecular Graphics System, Version 1.7.4 Schrödinger, LLC.

**Supplementary References**

1. Umeda, T. *et al.* Molecular basis of fosmidomycin’s action on the human malaria parasite *Plasmodium falciparum*. *Sci. Rep.* **1,** 9 (2011).

2. Trott, O. & Olson, A. J. AutoDock Vina: improving the speed and accuracy of docking with a new scoring function, efficient optimization, and multithreading. *J. Comput. Chem.* **31,** 455–61 (2010).

**Supplementary Table S1**

| **Cell line** | **FR-900098 IC_50_ (nM)** |
| --- | --- |
|
|  |  |
| *P. falciparum* 3D7 (pan-sensitive, lab adapted) | 511.0 ± 50.4 |
| *P. falciparum* 7G8 (quinine, chloroquine, and pyrimethamine resistant) | 716.8 ± 30.7 |
| *P. falciparum* D6 (mefloquine resistant) | 727.0 ± 86.1 |
| *P. falciparum* D10 (mefloquine resistant) | 621.2 ± 59.1 |
| *P. falciparum* K1 (chloroquine and sulfadoxine-pyrimethamine resistant) | 493.9 ± 73.1 |
| *P. falciparum* IPC 5202 (chloroquine and artemisinin resistant) | 526.3 ± 44.5 |
| Supplementary Table S1. FR-900098 is active against multidrug resistant *P. falciparum*. | |

**Supplementary Figures**

**Supplementary Figure S1. ERJ-207 docked into PfDxr.** ERJ-207 was docked into the PFDxr crystal structure (grey; PDB 3AU8) in the presence of Mn^2+^ (purple) and NADPH (pink) using AutoDock VINA. ERJ-207 binds in three distinct conformations, each represented by poses shown in A, B and C. The conformations displayed in panels A and B are similar to the dominant orientation predicted for FSM binding. However, docking studies demonstrate that ERJ-207 may bind in an alternative conformation with its phosphate group (orange/red) located on the opposite side of the binding pocket (C), similar to the alternate binding mode observed for FSM (data not displayed).

**Supplementary Figure S2. Growth of *P. falciparum* treated with either EU-131, ERJ-207, or FR-900098 is rescued by IPP supplementation.** *P. falciparum* strain 3D7 was treated with EU-131 (A; triangles), ERJ-207 (B; circles), or FR-900098 (C; squares) at a range of concentrations and growth quantified by PicoGreen (Life Technologies) after 72 h. The downstream isoprenoid precursor IPP rescues parasite growth (open shapes) indicating the inhibitors target the MEP pathway in *P. falciparum*. Shown are representative graphs from 3 or more independent experiments.

**Supplementary Figure S3. *P. falciparum* strains with high levels of the Dxr substrate DOXP confer resistance to EU-131, ERJ-207, and FR-900098.** Dose-dependent growth inhibition by EU-131 (A), ERJ-207 (B), or FR-900098 (C) was determined for the parental strain (3D7; closed circles, grey line), the FSM-resistant PfHad1 loss-of-function parasite strain (*had1*; open squares, black line), and the FSM-sensitive complemented strain (*had1* + HAD1-GFP; closed squares, black line). For EU-131, the parent strain has an IC_50_ = 51.6 ± 3.9 nM, while *had1* has an IC_50_ = 412.0 ± 20.5 nM. For ERJ-207, the parent strain has an IC_50_ = 202.8 ± 15.2 nM, while *had1* has an IC_50_ = 662.9 ± 74.7 nM. For FR-900098, the parent strain has an IC_50_ = 511.0 ± 50.4 nM, while *had1* has an IC_50_ = 2143.3 ± 366.3 nM. Data (mean ± SEM) are representative of at least 3 independent biological replicates performed in duplicate.

**Supplementary Figure S4. Neither EU-131 nor ERJ-207 eliminate mature gametocytes.** *P. falciparum* gametocytes were purified on a Percoll gradient and then treated for 72 h with either 5 μM EU-131 or ERJ-207. Parasites were stained with the mitochondrial dye MitoProbe DiIC(1)5 and % viability quantified by flow cytometry. 32.5 nM epoxomicin and 0.5% DMSO were used as positive and negative controls, respectively. While epoxomicin reduced gametocyte viability (p < 0.01), EU-131 and ERJ-207 were not significantly different than the DMSO control. Displayed are the means ± SEM from at least 2 independent experiments.

**Supplementary Figure S5. RCB-185 is ineffective against mid-stage and late-stage gametocytes.** *P. falciparum* gametocytes were purified on a Percoll gradient and then treated for 72 h with RCB-185 at concentrations ranging between 1 nM – 5 μM. Parasites were stained with MitoProbe DiIC(1)5 and % viability quantified by flow cytometry. Data indicate that, unlike the gametocytocidal compound epoxomicin (Fig. 5), RCB-185 does not inhibit mid-stage (A) or late-stage (B) gametocytes. Data were normalized to either the lowest treatment concentration (1 nM) (A) or the DMSO control (B). Triplicate data (mean ± SEM) are displayed from three independent samples at each treatment concentration.

**Supplementary Figure S6. Final synthetic step required for Dipivaloyloxymethyl 3-(N-(hydroxyl)acetylamino)-1-propenylphosphonate (RCB-185).** To a stirred solution of the benzyl starting material (1.4 g, 2.7 mmol) and pentamethylbenzene (2 g, 13.6 mmol) in dry dichloromethane (20 mL) under a nitrogen atmosphere at -78°C was added borontrichloride (1M in Hexane, 13.6 mL, 13.6 mmol) dropwise. The reaction was monitored by TLC. After 1 hour, the reaction mixture was poured into saturated aqueous sodium bicarbonate, and the aqueous layer was extracted with dichloromethane. The organic layers were combined and washed with brine, then dried over MgSO_4_ and the solvent was removed under reduced pressure. The crude oil was purified on a silica gel column using ethyl acetate and dichloromethane, giving the desired compound (594 mg, 51%) as a light yellow oil. ^1^H NMR (CDCl_3_, 399.79 MHz), δ (ppm): 8.61 (s, 1H), 6.71-6.84 (m, 1H), 5.86-5.96 (t, 1H), 5.61-5.69 (m, 4H), 4.41 (s, 2H), 2.19 (s, 3H), 1.22 (s, 18H). ^13^C NMR (CDCl_3_, 100.52 MHz), δ (ppm): 20.47, 26.93, 38.88, 50.30 (d, *J* = 26.6 Hz), 81.70 (d, *J* = 5.32 Hz), 117.95 (d, *J* = 189.17 Hz), 148.50, 172.87, 177.30. LCMS (ESI) *m/z*: 446 (M+Na), 847 (2M+1), 869 (2M+Na).
